# Can the provision of sexual healthcare for oncology patients be improved? A literature review of educational interventions for healthcare professionals

**DOI:** 10.1007/s11764-020-00898-4

**Published:** 2020-06-01

**Authors:** L.F. Albers, L.A. Grondhuis Palacios, R.C.M. Pelger, H.W. Elzevier

**Affiliations:** 1 Department of Urology, Leiden University Medical Centre, PO-box 9600, 2300 WB Leiden, The Netherlands; 2 Department of Medical Decision Making, Leiden University Medical Centre, PO-box 9600, 2300 WB Leiden, The Netherlands

**Keywords:** Sexual health, Education, Oncosexology, Quality of life, Quality of care

## Abstract

**Purpose:**

Sexual health is an important quality-of-life concern for cancer patients and survivors, but a difficult discussion topic for patients and healthcare professionals. The most important barriers causing healthcare professionals to avoid the topic are lack of education and lack of knowledge. How effective education about sexual health is for oncology healthcare professionals is not clear. The aim of this review is to examine the effectiveness of interventions in improving the provision of sexual healthcare for cancer patients.

**Methods:**

A systematic literature review was conducted according to PRISMA guidelines using the following data sources: PubMed, PsychInfo, Embase and Emcare. Quantitative research was included which contained pre-intervention and post-intervention outcomes. The assessment of the studies was conducted independently by two reviewers. A third reviewer was involved if there was no consensus.

**Results:**

Seven studies were included. In total, 572 oncology healthcare professionals participated, including physicians, nurses and allied healthcare professionals. Interventions consisted of 6 face-to-face sessions and one online program. Primary objectives of the studies were the assessment of improvement in knowledge about sexual health, improvement of practice, frequency of discussing sexual health and comfort level and the decline of perceived barriers to discussing sexual health. Studies showed that interventions resulted in improved realization of the objectives.

**Conclusions:**

Although improvement in the knowledge of healthcare professionals was achieved, it was not possible to give an overall recommendation for the development of interventions due to the limited number of studies and heterogeneity of the data.

**Implications for Cancer Survivors:**

Sexual health is an important area of survivorship that is often neglected. Many oncology healthcare professionals lack training and knowledge to provide such care. More evidence-based practices are needed to improve sexual healthcare for cancer survivors.

**Electronic supplementary material:**

The online version of this article (10.1007/s11764-020-00898-4) contains supplementary material, which is available to authorized users.

## Introduction

Sexual health is an important quality-of-life issue in cancer patients and survivors. The negative effect of cancer and its treatment on sexual health are widely described in the literature [1–12]. Sexual side effects can affect patients regardless of age, gender or cancer site. All treatment modalities, surgery, chemotherapy or radiotherapy cause specific sexual problems and can, therefore, impair sexual health. These problems might arise at the beginning of treatment; it is likely they will continue during long-term follow-up and survival [4, 10, 13–16]. Hence, the probability is that all healthcare professionals working with cancer patients will encounter patients who experience sexual problems as a result of their disease or treatment.

Cancer patients and survivors report a need for more information and support regarding sexual health issues [15, 17, 18]. They prefer to discuss sexual health with a healthcare professional that they expect to initiate the topic [13, 19, 20]. However, communication about sexual health in oncology care is reported to be challenging [21, 22]. Although healthcare professionals do feel a responsibility to discuss the subject, literature reveals that such discussions between patient and professional are limited [13, 21–23]. Healthcare professionals experience various barriers to discussing the subject; those most commonly reported are lack of knowledge and lack of training [21, 24–34]. Current literature highlights the need for more training and educational interventions for healthcare professionals to enhance patient-professional communication about sexual health [24, 25, 28–32, 35, 36].

Given these literature recommendations, we aimed to explore which educational interventions for oncology healthcare professionals, designed to enhance the provision of sexual healthcare for oncology patients, have so far been studied and how effective they are. The results of this review could inform the development and implementation of new interventions.

## Methods

This review was performed following the Preferred Reporting Items for Systematic Reviews and Meta-Analyses (PRISMA).

### Search strategy and outcome

We conducted a comprehensive literature search in PubMed, PsychInfo, Embase and Emcare with the help of a professional science librarian. The final search included three sets of search items (see supplement 1 for the full search) in the title or abstract linked with “AND”, pertaining to (a) oncology (neoplasma, cancer, adenoma, malignancy), (b) sexual health (sexuality, sex counselling, sexual behaviour, sexual dysfunction) and (c) education (workshop, training, physicians’ discussion).

Eligibility criteria applied for study inclusion are listed in Table 1. Studies in which the intervention group was compared to either a control group or baseline were included. We had no time restriction since no previous review of this topic was available. The initial search yielded 1171 studies. First, titles and abstracts were screened for eligibility criteria by two authors (LA and LG). If the article was selected, the full text was screened. Consensus discussions involved a third author (HE) if doubts about inclusion existed.

**Table 1:** Eligibility criteria for inclusion of studies

Items	Eligibility criteria
Participant	All healthcare providers who work with oncology patients
Study design	Quantitative interventions study
Language	English
Date of search	No limitation
Type of intervention	All educational/training interventions for healthcare providers with the aim of enhancing provision of sexual healthcare to oncology patients
Type of outcome	Studies reported at least one pre-intervention measurement and one a post-intervention measurement

**Fig. 1:**
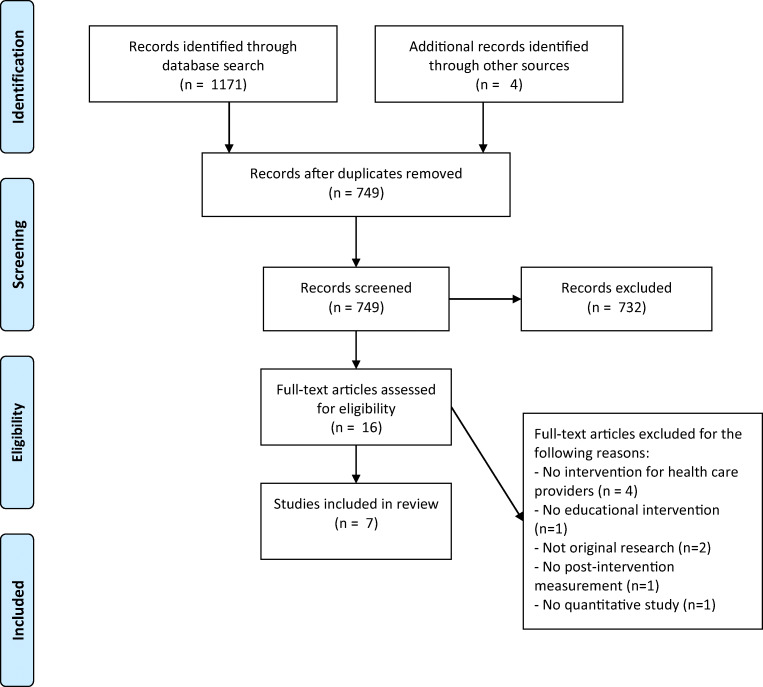
PRISMA flow diagram

After screening for title and abstract, 16 full-text articles were assessed for eligibility. Finally, 7 studies were included in the review (see flow diagram in Fig. 1).

### Level of evidence and quality appraisal

Level of evidence, based on the Oxford Centre for Evidence-Based Medicine guidelines, was assigned using the levels of evidence rating system [37], the scale ranging from 1 to 5. Level 1 represents a meta-analysis of randomized controlled trials (RCTs) or a systematic review; level 2 an RCT; level 3 a non-randomized controlled cohort/follow-up study; level 4 a case-series, case-control, or historically controlled studies; and level 5 a mechanism-based reasoning.

The Joanna Briggs Institute Critical Appraisal tools were used for quality appraisal of the studies (see Supplement 2).

Two reviewers (LA and LG) scored the studies independently. If no consensus was reached, a third reviewer (HE) was involved. No study was excluded on the basis of the assessment.

### Data abstraction

Data was extracted by the first author using a standardized coding sheet (Tables 2 and 3) and verified for correctness by a second author (LG).

**Table 2:** Brief overview of studies included

Source	Intervention type	Sample	Patient type	Work setting	Country	Follow-up	End response	Level of evidence
Pre−/post-questionnaires without control group								
Hordern (2009)	Single workshop (4.5 h)	155 oncology nurses and allied HCPs	Not specified	Unknown	Australia	Immediately post-workshop, 8 weeks	58.6%	3
Wang (2015)	Single training (30–45 min)	9 oncology physicians, 62 nurses/allied HCPs	Breast cancer	Suburban, four-hospital healthcare system	USA	Three to 6 months	50%	3
Afiyanti (2016)	Five days’ training (35 h)	46 oncology nurses	Not specified	Hospitals specialized in cancer services	Indonesia	Three weeks	100%	3
Jonsdottir (2016)	Comprehensive long-term educational intervention project (2 years)	210 oncology nurses and physicians	Not specified	University hospital	Iceland	10 months, 16 months	38%	3
								3
Grondhuis (2019)	One symposium (1 day)	55 uro-oncology HCPs	Prostate cancer	Various	The Netherlands	Six months	75%	
Pre-/post-questionnaires with randomized control group								
Kim (2014)	Eight e-learning sessions (total 16 h, 8 weeks)	31 oncology nurses (15 interventions, 16 controls)	Not specified	Tertiary hospital	Korea	Three months	100%	2
Mixed methods: pre-/post-questionnaire and audio records								
Reese (2019)	One self-study module (15 min), one workshop (60 min)	5 oncologists, 1 nurse practitioner, 1 physician assistant 134 breast cancer patients	Breast cancer	Cancer centre	USA	Healthcare professionals: direct post-intervention, 1 month, 6 months Patients: immediately after the visit	100%	3

**Table 3:** Overview of the interventions

Jonsdottir (2016)	Intervention type	Hospital-wide educational intervention project lasting 2 years to integrate sexual health into oncology, consisting of: - Identification of a team of 25 ‘change agents’ who act as role models on their wards - Establishment of a sexuality counselling service for cancer patients - Education and training of staff (40 staff members from 10 different units): two 5-h workshops focused on attitudes and practices. Teaching methods applied were lectures, group discussion, taking sexual history. The second workshop focused on more role play exercises to practice communication - Educational meetings between staff and (ward) change agents (20–30 min), about communication strategies; practical issues and screening possibilities were discussed - Development of a staff pocket-guide for nurses and physicians as an aid to initiate communication - Development of patient information material - Development of a website about cancer and sexuality for healthcare providers and patients
	Measurement	Self-report questionnaire, enquiring about: practice issues (8, 5-point Likert scale), attitudes (8, 5-point Likert scale), frequency of discussing topic (1, multiple choice), barriers (1, multiple choice), responsibility for initiative (1, multiple choice)
	Outcomes	- Change in mean scores before the intervention and at 16 months - Knowledge and training (1), practices issues (2), frequency of discussing topic (3), initiative (4), barriers (5)
	Results	(1) Have acquired sufficient knowledge and training; resp. *p* = 0.01, *p* = 0.006 (2) 5/8 practice issues improved; *p* < 0.01 (3) No change in frequency of discussing topic (4) No change in initiative (5) Fewer perceived barriers; *p* = 0.038
Kim (2014)	Intervention type	- Online problem-based learning (e-PBL); case videos with eight tutorials involving sexual healthcare problem scenarios; one session presented each week (1–2 h). - Posting solutions to the scenarios and discussions with others. - Additional online tools, such as video lectures, chat, discussion forum, databases, external website links were available
	Measurement	Self-report questionnaire containing: ‘Sexual healthcare knowledge scale’ (33, yes/no), ‘Sexual healthcare attitude scale’ (17, 3-point Likert scale), ‘Sexual health practice scale‘(21, yes/no)
	Outcomes	- Change in mean change for scores between intervention and control group at 3 months’ follow-up - Knowledge (1), attitude (2), practice (3)
	Results	(1) Higher knowledge score; *p* = 0.04 (2) No change in attitude score (3) No change in practice score
Wang (2015)	Intervention type	Single session, face-to-face, targeted sexual health training, 30–45 min. Traditional didactic education and communication skills training via brief role play and introduction of a user-friendly sexual health assessment tool
	Measurement	Self-reported questionnaire, enquiring about: comfort level (2, 5-point Likert scale), frequency (6, 5-point Likert scale), access to sexual health resource (1, 5-point Likert scale)
	Primary outcomes	- Changes in mean Likert scores between baseline and 6 months’ follow-up - Comfort level (1), self-reported frequency of addressing sexual issues (2)
	Results	1. Higher comfort level; *p* < 0.0001 2. Higher frequency of addressing issues; *p* < 0.0001
Reese (2019)	Intervention type	Single session self-study via information workbook (15 min) and single session workshop (90 min); skills-based, engagement in the first two steps of PLISSIT framework
	Measurement	Healthcare providers:- Self-reported questionnaire enquiring about: self-efficacy (3, 11-point scale), expected outcome regarding communication (7, 11-point scale), perceived barriers (14, 6-point scale) - Audio recording of clinic encounters Patients: - Satisfaction Index (4, 5-point Likert scale)
	Primary outcomes	Healthcare professionals: - Changes in mean scores between baseline and 6 months - Self-efficacy (1), outcome expectation (2), perceived barriers (3) - Odds/rate ratio; - Requesting/offering information about sexual health (4), complex issues involved in requesting/offering information (5), raising the topic(6), duration of sexual health communication(7) Patients: - Changes in mean score, between baseline and immediately after the consultation- Satisfaction (8)
	Results	(1) Increased self-efficacy; d = 0.27 (2) Increased outcome expectation; d = 0.69 (3) Reduced barriers; d = − 0.14 (4) Increased frequency of requesting/offering information; OR = 1.66/1.44, respectively (5) Increased complexity; OR = 1.65 (6) Increased frequency of raising the topic; OR = 2.38 (7) No change in duration; RR = 1.04 (8) No change in patient satisfaction
Grondhuis (2019)	Intervention type	One-day symposium with lectures on sexual dysfunction following several types of prostate cancer treatment and two workshops focusing on counselling techniques and tools to address sexual dysfunction in uro-oncological patients
	Measurement	Self-reported questionnaire (different for doctors, nurses/PAs, sexologists), enquiring about: knowledge (5-point Likert scale), discussion of sexual dysfunction (5-point Likert scale), rate of referral (5-point Likert scale), competence (3 polar questions: discussion of sexual function, advising on SD and actively enquiring about sexual issues
	Primary outcomes	- Changes in mean between baseline and six-months’ post-intervention - Knowledge (1), competence (2), frequency (3), referral rate (4)
	Results	(1) No change in knowledge; *p* = 0.39 (2)No change in competence; *p* = 0.25 (3) Higher frequency; *p* < 0.01 (4) No change in referral rate; *p* = 0.75
Afiyanti (2016)	Intervention type	Five-day competency-based training, 35 h in total, consisting of 6 sessions in the classroom or 3 days of lectures and 4 practice sessions. After the training, a 3-week mentorship process
	Measurement	Questionnaire including knowledge test (13 items, each with 5 answer options), and addressing attitudes/belief (14, 5-point Likert scale), self-efficacy (5, 5-point Likert scale), practice (11, 5-point Likert scale)
	Primary outcomes	- Changes in mean between baseline and 3 weeks post-intervention - Knowledge (1), attitude/belief (2), self-efficacy(3), practice(4)
	Results	(1) Higher knowledge score; *p* < 0.001 (2) Higher attitude/belief score; *p* = 0.008 (3) Higher self-efficacy score; *p* = 0.017 (4) No change in practice; *p* = 0.062
Hordern (2009)	Intervention type	Single-session, face-to-face workshop (4.5 h) with a professionally trained actor in the role of cancer patient to practice communication. The participants received feedback from the group
	Measurement	Self-reported questionnaire, addressing: barriers (20, 5-point Likert scale), confidence (7, 5-point Likert scale), practice (8, 5-point Likert scale)
	Primary outcomes	- Changes in means scores between baseline and 8 weeks’ follow-up - Barriers(1), confidence (2), practice (3)
	Results	(1) 16/20 barriers decreased; *p* < 0.01 (2) 7/7 confidence issues increased; *p* < 0.001. There were no significant effects of age or work experience on the participants’ confidence scores (3) 8/8 practice items increased; *p* < 0.003

## Results

### Participants

A total of 572 oncology (range 7–210) healthcare professionals participated in the seven included studies (Table 2). Of these, 556 healthcare professionals participated in an intervention; the other 16 acted as controls in one [38]. The participants included 384 nurses and other allied healthcare professionals, 48 physicians and 9 sexologists. The function of 131 participants, either oncologist or nurse, was not specified [27, 39]. Two studies focused specifically on healthcare professionals working with breast cancer patients and one on healthcare professionals working with prostate cancer patients [40–42]. The other studies did not specify an area of expertise of the participants.

### Design and quality appraisal

One study was a randomized control trial with a control group [38]. Six studies had a pre-post-questionnaire design without a control group [27, 39–43]. Of these six, one study described additional audio records of consultations between healthcare professional and patients (mixed-methods approach). The audio recording of clinic encounters was transcribed and coded for analysis. In addition, patients completed a questionnaire about the conversation with the healthcare professional immediately after the visit [40]. The time of follow-up varied between directly after the intervention and up to 16 months later. All study designs are described in Table 2. The quality appraisal showed very similar results in all studies (see supplement 2). The most common weakness was the lack of a control group.

### Type of interventions

A detailed overview of the interventions studied is presented in Table 3. The interventions used a combination of (video) lectures, symposia group discussions and practical sessions All interventions used in the studies were different and were developed by the authors or institution themselves. The duration of the intervention varied between 30 min and a 2-year programme. Four studies provided the healthcare professionals with a single-session intervention [39–42]. One study investigated a programme of 5 days [43]. Another study investigated hospital-wide multiple interventions over a period of 2 years [27]. Finally, one study evaluated eight online tutorials for a period 8 weeks. This was the only fully online intervention [38].

### Type of measurement

Self-reported questionnaires were used in all studies to evaluate outcome pre- and post-intervention [27, 38–43]. Only Kim et al. used questionnaires which had previously been described in literature and had proved to be valid and reliable [38, 44]. The questionnaires used in the other studies were developed by the authors based on social cognitive models, guidelines, previous studies, literature or expert opinion. They contained questions about knowledge, attitude, practice patterns, perceived barriers and comfort level. In addition, one study assessed clinical communication coded from audio-recorded conversations, patient satisfaction via a questionnaire and the duration of sexual health communication [40]. All measurements are summarized in Table 3.

### Objectives and results

Most primary objectives were described as the assessment of having acquired sufficient knowledge about sexual health, improvement of practice, frequency of discussing sexual health and comfort level and the decline of perceived barriers to discussing sexual health. All objectives and results are displayed in Table 3.

Three studies measured the perception about having acquired sufficient knowledge and training to be able to discuss sexual health [27, 38, 41]. Two studies reported a significantly higher self-reported knowledge score after the intervention [27, 38]. The interventions in these two studies contained multiple education moments, in contrast to the study without an effect [41]. Participants of one study performed a test which assessed their knowledge about sexual health, before and after the intervention. Participants scored significantly higher after the intervention [43].

Four studies measured current practices, such as giving patients oral or written information about sexual health, initiating discussions and referrals to another professional [27, 38, 39, 43]. Of these studies, two showed no significant improvement in practice [38, 43]. One of these investigated an online intervention with no face-to-face contact [38]. The other study had a 35-h programme over a period of 5 days [43].

The frequency of discussing sexual health was measured in four studies [27, 40–42]. In three, the frequency increased. The study which did not find this effect had a longer follow-up time (16 months) compared to the others (6 months) [27].

Three studies described the effect of the intervention on perceived barriers to discussing sexual health, such as lack of time, privacy, difficult topic to discuss, embarrassment and fear patient will react negatively. All showed a significant decrease in perceived barriers [27, 39, 40].

Six studies described a comfort level score for discussing sexual health (e.g. confidence, attitude or self-efficacy level) [38–40, 42, 43]. The five studies which showed a significant effect were skill-based interventions [39, 40, 42, 43].

One study assessed the patients’ satisfaction and length of the total consultation [40]. Patient satisfaction did not change significantly over time, nor did the duration of the total conversation. Most sexual health discussions lasted less than 1 min.

### Consent, completion and feedback from the participants

The acceptance rate for participation described in two studies was 50% and 88% [40, 42]. Reasons for non-participation were not described. All studies described completion of the intervention and questionnaire. The rate of completion ranged from 38 to 100% [27, 38–43]. In terms of acceptability and feasibility of the programmes, participants in four studies returned feedback about the intervention [27, 38, 40, 42]. Content of the intervention was considered as useful and relevant for the area of practice [27, 42]. Two studies described a level of satisfaction with the intervention of 53% and a score of 4.1/5 [38, 40].

## Discussion

In this systematic review, we identified studies which evaluated educational interventions for oncology healthcare professionals to improve communication about sexual health with patients.

Healthcare professionals may benefit from these educational interventions. These studies found an increase in the number reporting having sufficient knowledge, frequency of discussing, comfort levels and fewer perceived barriers due to an intervention for healthcare professionals. The results should, however, be interpreted with caution given the lack of control groups, small intervention groups, lack of validated questionnaires and absence of long-term follow-up.

We did not expect the studies to be so limited, given the large quantity of publications highlighting the need for education of healthcare professionals due to their frequently reported lack of knowledge and training. Unfortunately, it was not possible to provide an overall recommendation because of the heterogeneity of the data. The interventions, measurement, follow-up duration and outcomes were different in the included studies. Moreover, the most common weaknesses in the study design were the lack of a control group and the lack of long-term follow-up. As a result, long-term effect of the interventions is unknown. There is no indication on how frequently the interventions should be repeated for an optimal result.

The relationship between education and practice performance of clinicians has been widely studied. A review about this subject stated that “live, face-to-face educational activities are effective, especially when combined with multiple exposures to the information following the live educational activity [45].” Besides, multiple educational techniques have a greater long-term effect on practice performance than a single technique. Multiple exposures also have a favourable effect on the performance [45]. Against this background, the comprehensive long-term education programme of Jonsdottir et al. meets these conditions [27]. Still, no changes were found in frequency of discussing sexual health or in taking the initiative to discuss the topic, between baseline and 16 months’ follow-up. This might be due to barriers perceived by the healthcare professionals or the fact that not all healthcare professionals might want to become an expert in discussing sexual health.

In our review, studies with face-to-face, skill-based interventions, for example a role play exercise during a workshop, showed a significant increase in comfort level of the participants to approach a discussion. Practicing during the interventions gives the participants the opportunity to apply their skills in a safe environment. The only online learning intervention did not show an increase in comfort level [38]. One might argue that face-to-face education with practice exercises is more effective for a taboo subject such as sexual health in overcoming feelings of shame, a frequently reported barrier to discussing sexual health with patients [25, 28, 31]. Also, a qualitative study which focused on feedback about an educational intervention designed to enhance communication about sexual health, described that a role play exercise boosts the courage of the participants to initiate conversation [46]. However, face-to-face interventions are mostly time-consuming. Time is an important consideration when developing a new intervention for healthcare professionals, as lack of time is already a barrier to discussing sexual health. The study by Wang et al. described a face-to-face, targeted, single sexual health training lasting 30–45 min [42]. Both comfort level and frequency of addressing the topic were increased after 6 months follow-up, indicating a brief training might be sufficient. This result should, however, be interpreted with caution as it was a pilot study with a small number of participants and a high attrition rate.

Thus, in order to integrate sexual healthcare into medical practice, more is needed than education for individual oncology healthcare professionals. Financial aspects and organizational factors, like clinical space and agreement that healthcare professionals will devote time to providing sexual healthcare, are also important [47]. Current literature lacks proof of the optimal format of sexual health in oncology care. A few studies investigated interventions, other than educational, to enhance sexual healthcare. A prospective observational cohort study assessed the impact of a screening tool, the ‘Brief Sexual Symptom Checklist for Women’, used by oncology healthcare professionals, on the referral rates to allied healthcare professionals, like sexual counsellor or psychologist. No significant difference in referral was found. Moreover, more than half of the patients failed to attend sexual counselling following referral by their specialist [48]. The effectiveness of a nursing record focused on sexual healthcare was tested among oncology nurses in a randomized control trial [49]. The record was based on the PLISSIT model, commonly used for clinicians to discuss sexual health. The use of the record had a significant effect on the sexual healthcare practice of nurses compared to the control group. There was, however, no difference in sexual healthcare attitude score (discomfort, feeling uncertain), which might indicate the need for additional skill-based training.

Another study which investigated a multidisciplinary sexual health programme implemented in their hospital faced different challenges, like lack of funding, lack of staff and excessive waiting times due to heavy use of the clinic [47, 50]. They found that basic resources were lacking; patients were not having their sexual health concerns addressed elsewhere during their treatment process [47]. They highlight the need for oncology healthcare professionals to address sexual health proactively and thus reduce referral to the programme. The need for support from the Department of Nursing and an inter-professional team approach were highlighted as important issues by these studies [47, 50]. A network of representatives from different departments, like psychiatry, social work and urology, is needed to assist with cases as required. They do not actually have to attend the sexual healthcare clinic in person but should be available for consultation if required [50].

Some limitations need to be considered. Only seven studies were included in this review. Most studies were small and did not have a control group. Selection bias may have occurred as in six studies, the participants were not randomized. Moreover, the recruitment of participants was by self-selection or not adequately described in most studies. Response bias may have occurred in some studies due to attrition rates. It is likely that the most motivated participants completed the follow-up.

The long-term effect of the educational programmes is not known since only short-term follow-up was described in the studies. Due to the different outcome measurements used, it was not possible to provide an overall recommendation. To improve the comparison of future studies, it would be helpful if validated questionnaires were routinely used and a control group included. It is recommended that future studies are longitudinal in order to access the learning effect and practice over time. It would be interesting to include non-educational intervention to find out whether other factors can also contribute to the enhancement of sexual healthcare for oncology patients. In this context, including patient-reported data about patient satisfaction and duration of sexual health communication would be helpful to demonstrate that an improvement in the effect of interventions translates into improved patient satisfaction and quality of life [40].

Sexual health is an important area of cancer survivorship. There is a demand for sexual healthcare by the oncology patients, but many oncology healthcare professionals lack training and knowledge to provide such care. This systematic review provides an insight into the existing interventions and education of oncology healthcare professionals and might be helpful for the development of new interventions and studies. An overall recommendation for the development of interventions could not be given due to the limited number of studies and heterogeneity of the data. Notwithstanding, one could argue that following the interventions, healthcare professionals become more aware of the importance of addressing sexual health. More evidence-based practices are needed.

## Electronic supplementary material

ESM 1 (DOCX 12 kb)

ESM 2 (DOCX 21 kb)
