# Supplementary material for: Can the provision of sexual healthcare for oncology patients be improved? A literature review of educational interventions for healthcare professionals
Source: J Cancer Surviv. 2020 Jun 1;14(6):858–66. doi: 10.1007/s11764-020-00898-4 (PMC7572328; doi:10.1007/s11764-020-00898-4)
Supplement: Supplementary file 1 — (DOCX 12 kb) [file 11764_2020_898_MOESM1_ESM.docx]

S1. Supplementary information.

Search in PubMed. Search date: 11 October 2019. Limitations: English language.

(neoplasms[majr] OR "Cancer Survivors"[majr] OR cancer*[ti] OR adenoma*[ti] OR adenocarcinom*[ti] OR anticarcinogen*[ti] OR antitumour*[ti] OR blastoma*[ti] OR carcinogen*[ti] OR carcinom*[ti] OR carcinosarcoma*[ti] OR chordoma*[ti] OR germinoma*[ti] OR gonadoblastoma*[ti] OR hepatoblastoma*[ti] OR hodgkin disease[ti] OR hodgkin's disease[ti] OR hodgkins disease[ti] OR leukemi*[ti] OR lymphangioma*[ti] OR lymphangiomyoma*[ti] OR lymphangiosarcoma*[ti] OR lymphom*[ti] OR malignan*[ti] OR maligne[ti] OR malignes[ti] OR melanom*[ti] OR meningioma*[ti] OR mesenchymoma*[ti] OR mesonephroma*[ti] OR metasta*[ti] OR neoplas*[ti] OR neuroma*[ti] OR nsclc[ti] OR oncogen*[ti] OR oncolog*[ti] OR paraneoplastic[ti] OR plasmacytoma*[ti] OR precancerous[ti] OR sarcoma*[ti] OR teratocarcinoma*[ti] OR teratoma*[ti] OR tumor[ti] OR tumors[ti] OR tumour*[ti]) AND ("Sexual Dysfunctions, Psychological"[majr] OR "Sexual Behavior"[majr] OR "Sexual Partners"[majr] OR "Sexual Dysfunction, Physiological"[majr] OR "Sexual Health"[majr] OR "Orgasm"[majr] OR "Coitus"[majr] OR sex[ti] OR sexual*[ti] OR orgasm[ti] OR orgasms[ti] OR erection[ti] OR erections[ti] OR erectile[ti] OR coitus[ti] OR intercourse[ti] OR "Sex Education"[majr] OR "Sex Counseling"[majr]) AND ("Education"[majr] OR educat*[ti] OR workshop*[ti] OR train[ti] OR training*[ti] OR trained[ti] OR program[ti] OR programs[ti] OR teach[ti] OR teached[ti] OR teaching[ti] OR intervention*[ti] OR course[ti] OR courses[ti] OR "Learning"[majr] OR learn[ti] OR learned[ti] OR learning[ti] OR knowledge[ti] OR "Role Playing"[majr] OR role play*[ti] OR quality improve*[tw] OR "improving quality"[tw] OR "improving the quality" [tw] OR supportive car*[tw] OR support car*[tw] OR physician discussion*[tw] OR physicians discussion*[tw] OR physician's discussion*[tw] OR (quality[ti] AND improv*[ti])) AND (English[lang] OR Dutch[lang])
333 (11 okt 2019)
