# Supplementary material for: Can the provision of sexual healthcare for oncology patients be improved? A literature review of educational interventions for healthcare professionals
Source: J Cancer Surviv. 2020 Jun 1;14(6):858–66. doi: 10.1007/s11764-020-00898-4 (PMC7572328; doi:10.1007/s11764-020-00898-4)
Supplement: Supplementary file 2 — (DOCX 21 kb) [file 11764_2020_898_MOESM2_ESM.docx]

S2.Supplementary information. Assessment of risk of bias in individual studies according to the JBI -MAStARI Critical appraisal checklist

| JBI -MAStARI Critical appraisal checklist (items 1-13) for randomized Controlled Trials | Kim, et al 2014  et al.,2009 | JBI -MAStARI Critical appraisal checklist for Quasi-Experimental Studies (items 1-9). | Jonsdottir et al, 2016 | Wang et al, 2015 | Reese et al,  2019 | Afityanti et al, 2016 | Hordern et al,  2009 | Grondhuis et al,  2019 |
| --- | --- | --- | --- | --- | --- | --- | --- | --- |
| 1. Was true randomization used for assignment of participants to treatment groups? | Y | 1. Is it clear in the study what the ‘cause’ is and what the ‘effect’ | Y | Y | Y | Y | Y | Y |
| 2. Was allocation to treatment groups concealed? | Y | 2. Were the participants included in any comparisons similar? | Y | Y | Y | Y | Y | N |
| 3. Were treatment groups similar at baseline? | Y | 3. Were the participants included in any comparisons receiving similar treatment/care, other than the exposure or intervention of interest? | Y | Y | Y | Y | Y | Y |
| 4. Were participants blind to treatment assignment? | N | 4. Was there a control group? | N | N | N | N | N | N |
| 5. Were those delivering treatment blind to treatment assignment? | N | 5. Were there multiple measurements of the outcome both pre- and post- the intervention/exposure? | Y | Y | Y | Y | Y | Y |
| 6. Were outcomes assessors blind to treatment assignment? | N | 6. Was follow-up complete and if not, were differences between groups in terms of their follow-up adequately described and analyzed? | Follow-up incomplete, although adequately described and analyzed | Follow-up incomplete, although adequately described and analyzed | Y | Y | Follow-up incomplete, although adequately described and analyzed | Follow-up incomplete, although adequately described and analyzed |
| 7. Were treatment groups treated identically other than the intervention of interest? | Y | 7. Were the outcomes of participants included in any comparisons measured in the same way? | Y | Y | Y | Y | Y | Y |
| 8. Was follow-up complete and if not, were differences between groups in terms of their follow-up adequately described and analyzed? | Y | 8. Were outcomes measured in a reliable way? | Y | Y | Y | Y | Y | Y |
| 9. Were participants analyzed in the groups to which they were randomized? | Y | 9. Was appropriate statistical analysis applied? | Y | Y | Y | N | Y | Y |
| 10. Were outcomes measured in the same way for treatment groups? | Y |  | | | | | | |
| 11. Were outcomes measured in a reliable way? | Y |  |  |  |  |  |  |  |
| 12. Was appropriate statistical analysis used? | Y |  |  |  |  |  |  |  |
| 13. Was the trial design appropriate, and any deviations from the standard RCT design (individual randomization, parallel groups) accounted for in the conduct and analysis of the trial? | Y |  |  |  |  |  |  |  |
